# Guías de práctica clínica: oportunidad para visibilizar la importancia de la medicina del laboratorio

**DOI:** 10.1515/almed-2020-0094

**Published:** 2021-04-13

**Authors:** Maria Santamaría González, Maria Ángels Ruiz Mínguez, María Monsalud Arrebola Ramírez, Xavier Filella Pla, María José Torrejón Martínez, Daniel Morell García, Miguel Ángel Castaño López, Juan Antonio Allué Palacín, María Dolores Albaladejo Otón, Nuria Giménez

**Affiliations:** Servicio de Bioquímica Clínica, Hospital Universitario Miguel Servet, Zaragoz, España; Comisión de Medicina de Laboratorio Basada en la Evidencia, Sociedad Española de Medicina del Laboratorio (SEQC-ML), Barcelona, España; Servicio de Medicina de Laboratorio, Fundació Hospital de l’Esperit Sant, Santa Coloma de Gramenet, Barcelona, España; Unidad de Gestión Clínica de Laboratorio, Hospital de la Axarquía (AGSEMA), Málaga, España; Servicio de Bioquímica y Genética Molecular (CDB), Hospital Clinic, IDIBAPS, Barcelona, España; Unidad de Gestión Clínica de Análisis Clínicos (UGC), Hospital Clínico San Carlos, Madrid, España; Servicio de Medicina de Laboratorio, Hospital Universitari Son Espases, Palma de Mallorca, España; Servicio de Bioquímica Clínica, Hospital Clínico Universitario Juan Ramón Jiménez, Huelva, España; Synlab Diagnosticos Globales, Sevilla, España; Servicio de Análisis Clínicos y Bioquímica, Hospital General Universitario Santa Lucía, Cartagena, España; Unidad de Investigación, Fundación para la Investigación, Mutua de Terrassa, Universidad de Barcelona, Barcelona, España; Laboratorio de Toxicología, Universitat Autònoma de Barcelona, Barcelona, España

**Keywords:** biomarcadores, guías de práctica clínica, medicina basada en la evidencia, laboratorio clínico, medicina de laboratorio

## Abstract

**Objetivos:**

Las guías de práctica clínica (GPC) son recomendaciones desarrolladas de forma sistemática para ayudar a profesionales y pacientes en la toma de decisiones sobre la atención sanitaria más apropiada. Destacan entre sus características que deben basarse en la evidencia científica y estar elaboradas por equipos multidisciplinares. El objetivo de este estudio fue evaluar, en GPC, el contenido de la información sobre aspectos propios del laboratorio clínico y la participación de los profesionales del laboratorio en su elaboración.

**Métodos:**

Se evaluaron 16 GPC recomendadas por la Sociedad Española de Medicina del Laboratorio (SEQC-ML) y/o seleccionadas en PubMed. En cada guía se evaluaron 80 aspectos relevantes relacionados con el laboratorio clínico y la autoría de profesionales del laboratorio.

**Resultados:**

Las 16 guías evaluadas contenían de media un 49% (DE: 11%) de información sobre los aspectos específicos analizados del laboratorio clínico. Por orden de mayor a menor frecuencia contenían, una información media de: 69% de las variables postanalíticas analizadas (DE: 20%), 52% de las preanalíticas (DE: 11%) y 43% de las analíticas (DE: 18%). Finalmente, la mitad de las guías incluían algún profesional del laboratorio en la autoría.

**Conclusiones:**

En las guías evaluadas fue frecuente observar carencias en la información sobre aspectos fundamentales del laboratorio clínico y únicamente la mitad de ellas incluía entre los autores a profesionales del laboratorio. Por ello, puede considerarse que todavía existe un margen de mejora amplio y sería recomendable una mayor incorporación de profesionales del laboratorio a los equipos multidisciplinares que las desarrollan.

## Introducción

Las guías de práctica clínica se definen como “un conjunto de recomendaciones basadas en una revisión sistemática de la evidencia y en la evaluación de los riesgos y beneficios de las diferentes alternativas, con el objetivo de optimizar la atención sanitaria a los pacientes” [1]. Representan una de las herramientas más importantes para optimizar la toma de decisiones clínicas [2].

Para la elaboración de las GPC es recomendable garantizar un equipo multidisciplinar que incluya a todos los estamentos y perfiles profesionales implicados en la atención del tema abordado, y que se utilice una metodología científica, rigurosa y explícita con la que establecer recomendaciones basadas en la mejor evidencia científica disponible [3]. En la práctica es frecuente observar cierta confusión entre los términos GPC y protocolo clínico. Los protocolos son documentos que recogen la secuencia lógica de actividades a desarrollar frente a un problema asistencial específico. Tienen un carácter más normativo que las GPC, surgen del consenso y tienen en cuenta los recursos del centro en que se van a aplicar [4], mientras las GPC coronan la cúspide de la pirámide jerárquica de evidencia [5].

Desde el año 2004, la Comisión de Medicina de Laboratorio Basada en la Evidencia de la Sociedad Española de Medicina del Laboratorio (SEQC-ML) se encarga de coordinar un grupo de trabajo y organizar y difundir las GPC que, a criterio de los expertos integrados en las diferentes Comisiones de la SEQC-ML, tengan un mayor impacto asistencial y/o sean de mayor calidad. De este modo se colabora en el acceso y divulgación de la información científica.

Durante estos años, al evaluar las GPC llama la atención la relativa frecuencia con que se han encontrado carencias importantes en relación con la calidad de la información sobre las pruebas del laboratorio. En un reciente artículo, en el que se evaluaron las GPC sobre cribado de cáncer de próstata con antígeno prostático específico (PSA), se encontró que sólo en el 9% de las GPC, el grupo elaborador contaba con profesionales del laboratorio clínico; y con frecuencia no se mencionaban aspectos importantes sobre las pruebas del laboratorio clínico [6]. Además, resulta sorprendente la frecuente ausencia de profesionales del laboratorio dentro de los equipos multidisciplinares que elaboran las GPC, situación que se produce incluso en aquellas donde las pruebas de laboratorio juegan un papel especialmente relevante para el manejo clínico.

El objetivo de este estudio fue evaluar en las GPC que contienen recomendaciones sobre pruebas de laboratorio aplicadas en el diagnóstico o seguimiento de una patología, el contenido de la información sobre aspectos propios del laboratorio clínico y el grado de participación de los profesionales del laboratorio.

## Materiales y métodos

### Fuentes de información y estrategias de búsqueda

Se realizó un estudio observacional descriptivo retrospectivo en el que se seleccionaron GPC de los últimos 5 años (2015–2019), a partir de dos fuentes de información: la plataforma de la Sociedad Española de Medicina del Laboratorio (SEQC-ML), consultada en diciembre de 2019, y el buscador bibliográfico PubMed.

Las GPC recomendadas por comisiones científicas de la SEQC-ML están disponibles con acceso libre en la web (http://www.seqc.es/es/gpc/).

En cuanto a la búsqueda bibliográfica, los autores consensuaron una estrategia limitada al laboratorio clínico y acotada a los últimos 5 años: ((“Guideline” [PublicationType] OR “Guidelines as Topic”[Mesh] OR guideline*) AND (“Clinical Laboratory Services”[Mesh] OR “Laboratories”[Mesh] OR laboratories)) AND (“Biomarkers”[Mesh] OR biomarkers) (búsqueda realizada el 29 noviembre 2019).

### Criterios de selección

Las GPC fueron seleccionadas para su inclusión si cumplían los siguientes criterios: tipo de publicación guía clínica, publicada en los últimos cinco años, escrita en inglés, y que la guía incluyese pruebas realizadas en el laboratorio clínico relevantes para el diagnóstico o tratamiento. Las GPC repetidas sólo se incluyeron una vez.

### Procedimiento de recolección de datos

La selección de las GPC la realizaron dos autores, de forma independiente, siguiendo los criterios de inclusión. Las discrepancias observadas se resolvieron por un tercer autor.

Las GPC seleccionadas fueron evaluadas por pares. Se recogieron aspectos relevantes del laboratorio clínico distribuidos entre las tres fases del proceso de análisis, que pueden afectar a la interpretación clínica de los resultados. Para ello se siguió el listado de comprobación de 80 aspectos relevantes en el laboratorio clínico propuesto por Aakre y colaboradores [7], adaptándolo a las GPC evaluadas. A cada variable del estudio se le otorgó una puntuación dicotómica de SI (1) o NO (0), siendo la mínima puntuación alcanzable de 0 y la máxima de 80.

Paralelamente se revisó la participación de profesionales del laboratorio en todas las GPC evaluadas, utilizando las afiliaciones de los autores. Se consideró dentro de este grupo a cualquier facultativo que desarrollara su actividad profesional dentro de un laboratorio ya fuese clínico o de investigación. Entendiendo como facultativo a todo profesional titulado superior, con formación específica, competente para ejercer una determinada función con responsabilidad personal final [8].

### Análisis estadístico

Los resultados se expresaron en porcentaje para las variables categóricas. Se estudió la normalidad con las pruebas de Kolmogorov-Smirnov y Shapiro-Wilk. Las variables continuas se expresaron con una medida de tendencia central (media o mediana) y una de dispersión (desviación estándar (DE) o rango intercuartil (RIC)), en función de su distribución. Las variables cualitativas se compararon utilizando el test de la *χ* ^2^. Para la comparación de las variables cuantitativas se utilizó el test de la «t» de Student o la prueba no paramétrica U de Mann-Whitney. En todas las comparaciones se utilizó un nivel de significación estadística de 0,05. Se utilizó el programa IBM SPSS versión 25 (Armonk, NY, USA).

## Resultados

**Figura 1:**
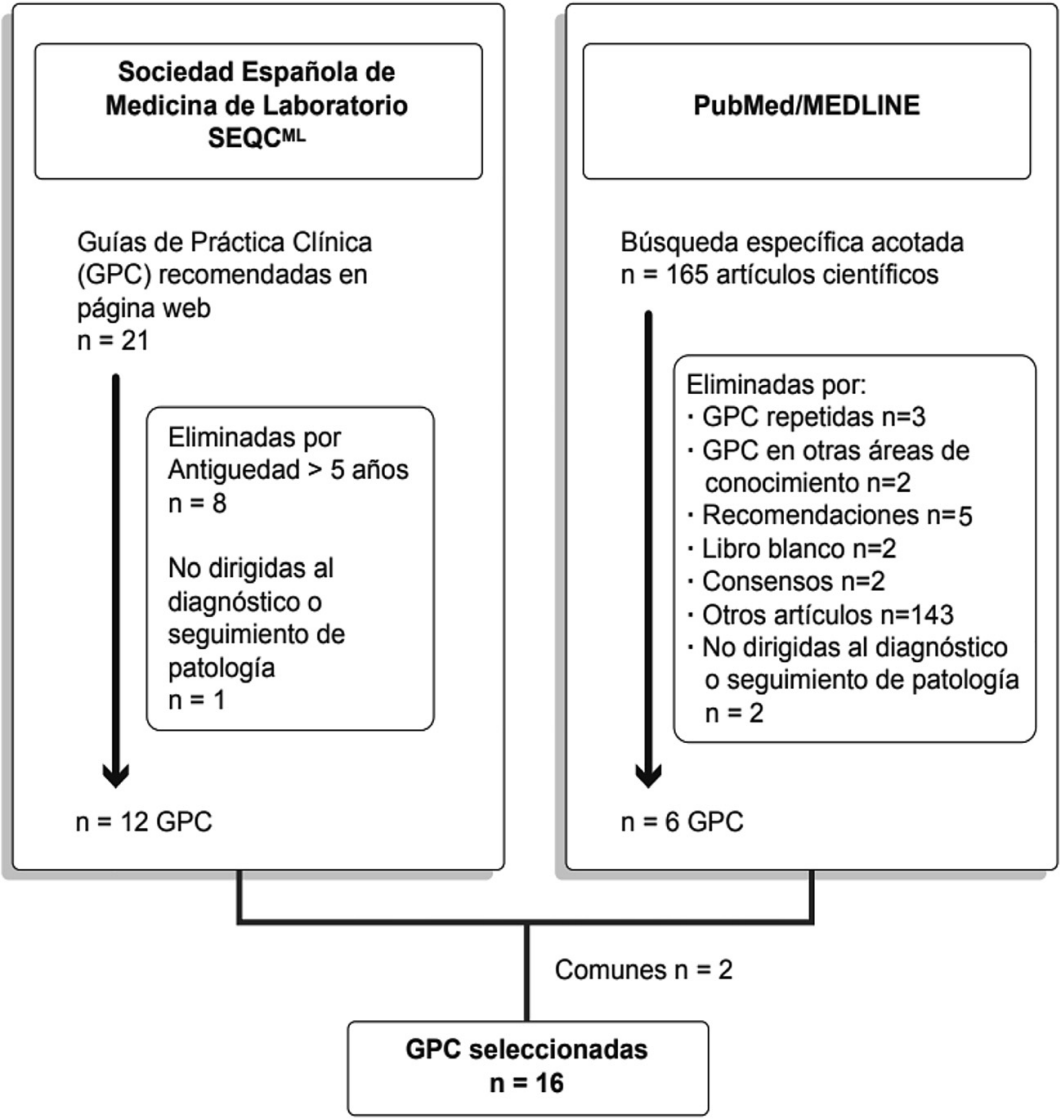
Selección de guías de práctica clínica.

El proceso de selección de las GPC se detalla en la Figura 1. La búsqueda bibliográfica en PubMed arrojó un total de 165 artículos científicos de los que se obtuvieron un total de 6 GPC tras aplicar los criterios de inclusión. Se excluyeron 143 artículos que sin pertenecer al tipo de publicación guía clínica hacían referencia a ellas. Asimismo, se cotejaron las 12 GPC recomendadas por la SEQC-ML, que cumplían los criterios de inclusión.

Finalmente, se obtuvieron un total de dieciséis GPC para su revisión y evaluación (Tabla 1).

**Tabla 1:** GPC evaluadas y porcentajes de las variables estudiadas que recogen aspectos importantes del laboratorio clínico en cada una de las fases preanalítica, analítica y postanalítica.

GPC evaluada (n=16) (máxima puntuación posible)	Fases del proceso analítico^a^				Profesional laboratorio n=16
	Preanalítica (33 puntos)	Analítica (38 puntos)	Postanalítica (9 puntos)	Total (80 puntos)	
Clinical use of cancer biomarkers in epithelial ovarian cancer: Updated guidelines from the European Group on Tumor Markers [9].	50% (16/32)	13% (5/38)	78% (7/9)	35% (28/79)	Sí
Clinical use of biomarkers in breast cancer: Updated guidelines from the European Group on Tumor Markers [10].	46% (12/26)	44% (11/25)	50% (3/6)	46% (26/57)	Sí
Revised American Thyroid Association Guidelines for the management of medullary thyroid carcinoma [11].	61% (17/28)	68% (15/22)	78% (7/9)	66% (39/59)	Sí
Congenital adrenal hyperplasia due to steroid 21-hydroxylase deficiency: An Endocrine Society Guideline [12].	66% (21/32)	68% (15/22)	67% (6/9)	67% (42/63)	No
Clinical practice guidelines for the care of girls and women with Turner syndrome [13].	42% (11/26)	73% (8/11)	60% (3/5)	52% (22/42)	Sí
European Guidelines on cardiovascular disease prevention in clinical practice [14].	48% (12/25)	52% (15/29)	56% (5/9)	51% (32/63)	No
Guideline on the management of blood cholesterol [15].	48% (16/33)	42% (16/38)	89% (8/9)	50% (40/80)	Sí
Guidelines for the management dyslipidaemias [16].	58% (19/33)	45% (17/38)	89% (8/9)	55% (44/80)	Sí
Guidelines for the diagnosis and treatment of acute and chronic heart failure [17].	27% (9/33)	18% (7/38)	56% (5/9)	26% (21/80)	No
Guidelines for the management of acute coronary syndromes in patients presenting without persistent ST-segment elevations [18].	76 % (22/29)	33 % (11/33)	89% (8/9)	58% (41/71)	No
Chronic kidney disease in adults [19].	42% (14/33)	39% (15/38)	89 % (8/9)	46 % (37/80)	No
Anaemia management in chronic kidney disease [20].	69% (20/29)	42% (13/31)	89% (8/9)	59% (41/69)	No
Updated molecular testing guideline for the selection of lung cancer patients for treatment with targeted tyrosine kinase inhibitors [21].	55% (18/33)	13% (5/38)	44% (4/9)	34% (27/80)	Sí
Biomarkers in non-small cell lung cancers: Indian consensus guidelines for molecular testing [22].	48% (14/29)	53% (17/32)	89% (8/9)	56% (39/70)	Sí
ESMO consensus guidelines for the management of patients with metastatic colorectal cancer [23].	45% (10/22)	37% (10/27)	22% (2/9)	38% (22/58)	No
Human epidermal growth factor receptor 2 testing in breast cancer [24].	48% (11/23)	48% (10/21)	56% (5/9)	49% (26/53)	No
**Totales**	52 %	43%	69%	49%	50% (8/16)

^a^Los resultados se han expresado como % (número absoluto de aspectos del laboratorio incluido/número absoluto de aspectos del laboratorio relevantes).

Todas las fases del proceso analítico estaban bien representadas en el conjunto de GPC evaluadas y contenían un 49% (DE: 11%) de media sobre los aspectos específicos del laboratorio clínico analizados. Por orden de mayor a menor frecuencia contenían una información media de: 69% (DE: 20%) de las variables postanalíticas analizadas, 52% (DE: 11%) de las preanalíticas y 43% (DE: 18%) de las analíticas.

La información contenida en las GPC sobre los aspectos del laboratorio de cada fase analítica se encuentra detallada en la Tabla 2, según autoría de profesionales del laboratorio y según la fuente de información a partir de la cual se seleccionó la GPC. En el 50% de las GPC evaluadas participaba algún profesional del laboratorio clínico dentro del grupo de elaboración. No se observó asociación entre la información sobre las fases analíticas contenida en las GPC y la autoría de profesionales del laboratorio (p=1,000) y tampoco se asoció a la fuente de información a partir de la cual se seleccionó la GPC (la web de SEQC-ML o de PubMed) (p=0,214) (ver Tabla 2).

**Tabla 2:** Información contenida en las guías de práctica clínica sobre las fases analíticas según autoría y fuente de información.

Información contenida en las guías de práctica clínica (% = número de aspectos del laboratorio detallados/número de aspectos máximos)				
GPC con autoría de profesionales del laboratorio				
n=16	Sí (n=8)		No (n=8)	p-Value
Fase preanalitica, media (DE)^a^	51(6)		53(16)	0,799
Fase analítica, media (DE)^a^	44 (22)		42 (15)	0,859
Fase postanalítica, mediana (RIC)^b^	78 (37)		62 (33)	0,705
Total proceso analítico, media (DE)^a^	49 (11)		49 (13)	1,000

**Fuente de información**				
**n=14^d^**		**SEQC-ML (n=10)**	**PubMed (n=4)**	**p-Value**

Fase preanalitica, media (DE)^a^		54 (15)	49 (4)	0,382
Fase analítica, media (DE)^a^		48 (17)	38 (18)	0,342
Fase postanalítica, mediana (RIC)^b^		84 (30)	50 (53)	0,089
Total proceso analítico, media (DE)^a^		53 (12)	44 (10)	0,214
Autoría de profesionales del laboratorio ^c^		40%	67%	0,298

DE, desviación estándar; RIC, rango intercuartil. ^a^Prueba T de Student, ^b^prueba no paramétrica U de Mann-Whitney, ^c^prueba de *χ* ^2^, ^d^se han excluido para los cálculos las dos GPC comunes para ambas fuentes de información.

La evaluación en porcentajes de cada uno de los 80 aspectos valorados en las GPC se resume en la Tabla 3. Los aspectos relacionados con los requerimientos de las muestras fueron los menos representados de la fase preanalítica y las interferencias analíticas incluidas dentro de la fase analítica las que menos se mencionaron en las GPC.

**Tabla 3:** Tabla utilizada para evaluar las GPC (modificada de Aakre et al. [7]).

Fase preanalítica		Inclusión en las GPC
Descripción de la población diana	Edad	88%
	Sexo	84%
	Enfermedad	96%
	Enfermedades específicas	94%
Indicaciones de uso del biomarcador	Monitorización	84%
	Frecuencia de las pruebas	68%
	Diagnóstico	83%
	Pronóstico	88%
	Cribado	57%
	Auto-monitorización	50%
Objetivos clínicos	Sensibilidad	65%
	Especificidad	59%
	Curva ROC	6%
	Valor añadido del biomarcador	81%
	Comparación con otros biomarcadores relacionados	69%
	Probabilidad de diagnóstico tras la prueba	52%
	VPP de la prueba	31%
	VPN de la prueba	28%
Enfoque múltiple con otros biomarcadores	Inclusión en un panel con otros marcadores	71%
	Sensibilidad (panel)	39%
	Especificidad (panel)	39%
	Curva ROC (panel)	0%
	Valor añadido del panel	57%
Requirimientos de muestra	Ayuno	35%
	Tiempo desde el evento clínico	28%
	Posición del paciente	5%
	Ritmo circadiano	10%
	Tipo de muestra	75%
	Transporte de la muestra	9%
	Centrifugación	0%
	Pretratamiento de la muestra (retraso máximo)	19%
	Tiempo máximo de almacenamiento a temperatura especificada	6%
	Número máximo de ciclos de congelación-descongelación	3%

Fase analítca		

Metodología	Método recomendado	72%
	Estandarización	41%
	Trazabilidad al método de referencia	38%
	Heterogeneidad de biomarcadores	50%
	Límite de detección	17%
Interferencias analíticas	Lipemia	6%
	Hemolisis	0%
	Bilirrubina	0%
	Paraproteínas monoclonales	6%
	Anticuerpos heterófilos	16%
	Autoanticuerpos endógenos	15%
	Factor reumatoide	6%
	Otros relevantes	30%
	Definir acciones cuando se sospecha interferencia analítica	10%
Interferencias biológicas	Edad	75%
	Sexo	68%
	Enfermedad aguda	70%
	Fase aguda	59%
	Ingesta de alimentos	42%
	Medicación	52%
	Tabaco	33%
	Alcohol	30%
	Embarazo	29%
	Postmenopausia	18%
	Obesidad	27%
	Actividad física	38%
	Factores genéticos	60%
	Etnia	37%
	Región geográfica	37%
	Otros	47%
Objetivos de calidad	Variabilidad analítica	53%
	Evaluación externa de la calidad	50%
	Evaluación interna de la calidad	44%
	Criterios/Objetivos de calidad	25%
	Sesgo, imprecisión y error total	22%
	Acreditación	28%
	Cualificación del personal	31%
	Tiempo de respuesta	31%

Fase postanalítica		

	Resultados cuantitativos o cualitativos	94%
	Unidades	87%
	Comentarios sobre resultados	56 %
	Intervalo de referencia	48%
	Punto de corte diagnóstico	90%
	Objetivo terapéutico	88%
	Variabilidad biológica	41%
	Información sobre cambios clínicos significativos (VRC)	37%
	Interpretación de cambios basados en VRC ó resultados de estudios clínicos	50%

Contiene 80 aspectos relevantes en el laboratorio clínico, repartidos entre la fase preanalítica, analítica y postanalítica. Si algún aspecto no se consideraba relevante para la evaluación de la GPC evaluada, el nominador se ha ajustado al calcular los porcentajes.

## Discusión

Este estudio muestra cómo pese a la indudable relevancia de las GPC para ofrecer una asistencia clínica adecuada, desde el laboratorio clínico todavía se dispone de un margen de mejora, tanto por las posibilidades de incrementar la participación en los equipos multidisciplinares que elaboran estas guías, como a la hora de mejorar la información contenida sobre aspectos específicos y concretos de las pruebas diagnósticas, tan fundamentales en el razonamiento y la toma de decisiones clínicas [25], [26], [27].

Se observó que las GPC que contienen recomendaciones sobre pruebas de laboratorio aplicadas en el diagnóstico o seguimiento de una patología recogían únicamente la mitad de la información relevante sobre aspectos fundamentales del laboratorio y algunos aspectos concretos como los requisitos preanalíticos de las muestras o las interferencias analíticas apenas se mencionaban, aunque en general, integraban todas las fases del proceso de análisis: preanalítica, analítica y postanalítica. Es importante conocer las limitaciones y los posibles errores que se pueden producir porque cualquier posible defecto en alguna de las fases puede tener un impacto negativo en el paciente [28]. Las pruebas de laboratorio son una parte esencial e integral en la toma de decisiones médicas y la mayoría de las GPC incluyen recomendaciones sobre el uso clínico de las pruebas de laboratorio. Sin embargo, la información sobre aspectos relevantes del laboratorio clínico suele ser incompleta [6, 7], a pesar de ser necesaria para una interpretación correcta del resultado [28]. La calidad de las GPC podría mejorar adecuadamente integrando un enfoque sobre aspectos relevantes del laboratorio [6, 7].

La mitad de las GPC estudiadas en este artículo incluían algún profesional del laboratorio en el proceso de desarrollo. Esta participación es inferior a la que sería deseable, puesto que se seleccionaron las GPC donde las pruebas de laboratorio eran especialmente relevantes para el diagnóstico o seguimiento de una patología. Sin embargo, los únicos estudios encontrados que analizan este aspecto obtienen incluso participaciones inferiores [6, 7]. En el grupo que desarrolla una GPC deberían participar todos los perfiles profesionales implicados en la atención del tema abordado, garantizando la multidisciplinariedad reconocida en las GPC. De este modo, la elaboración de las GPC que recomiendan el uso de pruebas de laboratorio sin involucrar a un especialista en medicina de laboratorio puede generar incertidumbre en la descripción de las pruebas de laboratorio [29]. Además, incluir la evidencia sobre la efectividad clínica de las pruebas conllevaría mejoras en las GPC, así como una utilización racional del laboratorio clínico y de los nuevos biomarcadores emergentes [25]. Es fundamental que los profesionales del laboratorio clínico se familiaricen con las GPC y consideren las limitaciones de todas las fases del proceso analítico para poder ofrecer consejo especializado y asesoramiento interpretativo a sus colegas clínicos [29]. La actividad de los profesionales de laboratorio está interconectada con todas las disciplinas médicas, y proporciona un soporte crucial en la atención sanitaria [27]. Por tanto, sería recomendable tomar conciencia de la importancia de promover la consultoría desde el laboratorio y la colaboración con todos los profesionales que participan en la asistencia a los pacientes. Además, sería aconsejable continuar potenciando la participación e implicación directa de los profesionales del laboratorio en los equipos multidisciplinares que elaboran las GPC, así como en su evaluación, difusión y aplicabilidad práctica.

En contraposición a lo esperado, en nuestro estudio no se ha observado que la inclusión de especialistas en medicina de laboratorio en los comités de desarrollo de las GPC evaluadas, conllevara un aumento general en la atención y cuidado de los aspectos relacionados con el laboratorio, al contrario de lo observado en otros estudios similares [7, 29]. Akree et al. describen que cuando en las GPC están implicados especialistas de medicina del laboratorio consta más información sobre el tipo de muestra, su transporte y los diversos aspectos analíticos [7]. También en la guía de la Sociedad Torácica Británica solicitan el apoyo de los profesionales de medicina del laboratorio para mejorar el uso e interpretación de los test del laboratorio por parte de los clínicos [29]. Consideramos posible que en nuestro estudio no hayamos encontrado diferencias estadísticamente significativas en la calidad de los aspectos relacionados con el laboratorio entre las GPC con y sin autoría de profesionales del laboratorio debido al método de selección. En general las GPC incluidas eran de excelente calidad, bien por haber sido recomendadas por los grupos de expertos que conforman las distintas Comisiones de SEQC-ML por su relevancia y utilidad para el laboratorio clínico o bien por la búsqueda acotada centrada en el laboratorio en PubMed. Nos parece factible, como hipótesis, que incluso en aquellas GPC sin autoría directa clara de profesionales de laboratorio, estos hayan intervenido como colaboradores externos, asesores, consultores expertos y/o revisores puesto que se incluyen muchos aspectos del área de conocimiento de la medicina del laboratorio.

Los facultativos del laboratorio son especialistas en las interacciones bioquímicas y biológicas que caracterizan a las patologías humanas. Su experiencia profesional sobre las pruebas analíticas y su utilidad clínica les proporciona una cualificación de consultores sobre los requisitos preanalíticos, y el valor semiológico e interpretación de los resultados analíticos, complementado así la información producida en los informes clínicos y garantizando la calidad del proceso asistencial en su totalidad [8]. Además, contribuyen a reducir los potenciales errores de diagnóstico y a mejorar la calidad de la prestación sanitaria y asistencial al paciente [30]. La medicina de laboratorio puede ser percibida ocasionalmente como una disciplina abandonada y un claro ejemplo son las numerosas publicaciones carentes de un conocimiento profundo de la importancia real de las pruebas de laboratorio [26]. Los profesionales del laboratorio clínico deben aumentar su visibilidad y contribuir con su participación activa en los equipos de atención clínica, generando consciencia colectiva de la experiencia y el valor añadido aportado como consultores de la atención centrada en el paciente.

La SEQC-ML, como sociedad científica, prioriza el acceso a información de alta calidad, recomendando GPC agrupadas por áreas de conocimiento. Además, estas guías se actualizan periódicamente, a fin de incorporar evidencia recientey dando respuesta a las necesidades de los profesionales del laboratorio clínico. La elevada cantidad de información biomédica disponible dificulta la actualización de los profesionales pues la búsqueda de artículos de mayor calidad es un proceso que consume tiempo y esfuerzo [31]. Disponer de un espacio web destinado a albergar guías clínicasrelevantes para el laboratorio facilita el proceso de selección a los especialistas [32]. De este modo, las sociedades científicas cumplen una de sus funciones básicas, gestionar el conocimiento y promover su creación, captación, difusión y manejo, así como favorecer la comunicación científica [33]. Además de esta iniciativa de publicar en la web oficial las GPC recomendadas por la sociedad científica, la SEQC-ML también promueve otras múltiples iniciativas, desde sus diferentes comisiones, para colaborar con otras sociedades científicas y visibilizar el trabajo de los profesionales del laboratorio.

Es importante destacar la existencia de ventajas y limitaciones al trasladar y aplicar las recomendaciones de las GPC a la práctica clínica [34], incluida la medicina de laboratorio. Una de las facilidades detectadas con este estudio es que la SEQC-ML habilita un espacio web de acceso fácil y rápido a las GPC, estando éstas organizadas y previamente revisadas. Existe acuerdo internacional sobre la necesidad de impulsar el uso de GPC entre los profesionales del laboratorio clínico y sobre los beneficios de implicarse en su elaboración y aplicación [35]. Sin embargo, una barrera que debemos considerar de forma evidente es la dificultad de los profesionales del laboratorio para acceder y formar parte de los equipos multidisciplinares que las elaboran.

Una posible limitación de este estudio fue el número reducido de GPC evaluadas. Se optó por recoger todas las GPC recomendadas por la SEQC-ML y se complementó con una búsqueda específica limitada a las GPC que trataban prioritariamente sobre aspectos relacionados con el laboratorio clínico. Por ello, debe entenderse que, a pesar del inevitable sesgo muestral en la selección de las GPC, la muestra utilizada resulta adecuada para los objetivos propuestos en este estudio.

## Conclusiones

La mayor aportación del presente estudio es resaltar la importancia que adquieren los profesionales del laboratorio y las sociedades científicas en su contribución al acceso y divulgación de la información científica, fomentando la colaboración entre grupos de expertos que recomiendan GPC con información relevante en cada campo de aplicación. Por todo ello recomendamos a los profesionales del laboratorio reforzar su papel consultor con el resto de profesionales y pacientes para visibilizar el funcionamiento de un laboratorio clínico, y fomentar la cooperación que permite evitar errores y mejorar la asistencia [36]. Al profesional del laboratorio le corresponde velar por el cuidado de todas las fases del proceso analítico, desde la fase analítica, la más evidente, que en principio concentraría la menor proporción de errores (13–32%), hasta las fases preanalítica y postanalítica, menos conocidas por el resto de los especialistas y pacientes [37].

Además, este estudio ha evidenciado puntos de mejora en la elaboración de las GPC. Estas podrían considerarse como un ejemplo de proyecto interdisciplinar donde cooperar y aportar los conocimientos de la especialidad y del área del laboratorio clínico que nos son propios. Participar en proyectos interdisciplinares aumentar la visibilidad de los profesionales del laboratorio. Sería conveniente, como futuro ámbito de estudio, analizar el grado de implementación de las GPC en la práctica diaria del laboratorio, su repercusión en la calidad asistencial y el compromiso de los profesionales con su elaboración y aplicación.
